# Safety of International Professional Sports Competitions During the COVID-19 Pandemic: The Association Football Experience

**DOI:** 10.1007/s40279-022-01763-3

**Published:** 2022-09-27

**Authors:** Horacio Caniza, Francisco Forriol, Osvaldo Pangrazio, Mario Gil-Conesa

**Affiliations:** 1 Universidad Paraguayo Alemana, San Lorenzo, Paraguay; 2 Universidad San Pablo-CEU, Madrid, Spain; 3 CONMEBOL Confederación Sudamericana de Fútbol, Luque, Paraguay; 4 Universidad de Navarra, Pamplona, Spain

## Abstract

Major sporting events were suspended during the most acute phase of the COVID-19 pandemic. Competitions are resuming with enhanced hygiene protocols and altered mechanics. While risks for players and staff have been studied, the impact of large-scale tournaments on the communities that host them remains largely unstudied. CONMEBOL Copa América is one of the first wide-scale international tournaments to be conducted in its original format since the beginning of the COVID-19 pandemic. The tournament saw 10 national teams compete in four Brazilian cities during a period of heightened viral transmission. The analysis of over 28,000 compulsory PCR tests showed that positive cases did not lead to the uncontrolled spread of the disease among staff and players. More importantly, the data indicate that locally hired staff were not exposed to increased risk while working. The Copa América experience shows that international sporting competitions can be conducted safely even under unfavourable epidemiological situations.

**Supplementary Information:**

The online version contains supplementary material available at 10.1007/s40279-022-01763-3.

## Key Points

**Table:** 

No uncontrolled spread of COVID-19 was detected in players or foreign and local staff during the CONMEBOL Copa América Association Football tournament conducted in four Brazilian cities in 2021.
Employment in Copa América did not increase risk to local contractors.
The Copa América experience shows that even in adverse epidemiological conditions, professional sporting competitions can be safe.

## Introduction

Association Football, like most professional sports, has been affected by the COVID-19 pandemic [1–3]. Training and competition have resumed with risk abatement protocols in place and changes to the mechanics of the tournament in an attempt to minimise risk.

Contact sports have the hallmarks of increased risk; nevertheless, the evidence shows these risks can be managed [4]. Meyer et al. describe the reopening of the German Bundesliga during a period of reduced transmission of the virus [5]. The authors concluded that football can safely restart following strict testing and hygiene protocols as they found no evidence of uncontained spread among players and staff. Schumacher et al., in a similar study, analysed the Qatari professional football league. The authors observed infection rates consistent with those of the general population, noting that infections seem to originate through social contacts rather than during the match [6]. In a notable exception, Gualano et al. observed increased risk for players in São Paulo [7].

Notably, the risks posed to the communities that host these tournaments remain largely unstudied. In this work, we analyse CONMEBOL Copa América 2020, a continental-scale Association Football tournament organised by CONMEBOL (Confederación Sudamericana de Fútbol, Confederação Sul-Americana de Futebol). After being postponed at the onset of the COVID-19 pandemic, 10 national teams with players from over 30 countries came together in Brazil between June and July of 2021. CONMEBOL implemented comprehensive protocols to minimise risks to the community and the more than 700 staff involved. We show that the protocols were able to isolate the tournament from the unfavourable epidemiological conditions in Brazil. The data also suggest that large-scale tournaments can be conducted safely, in such a way that they do not increase the risk for host communities.

## Methodological Aspects

### Accreditation and Location

**Fig. 1:**
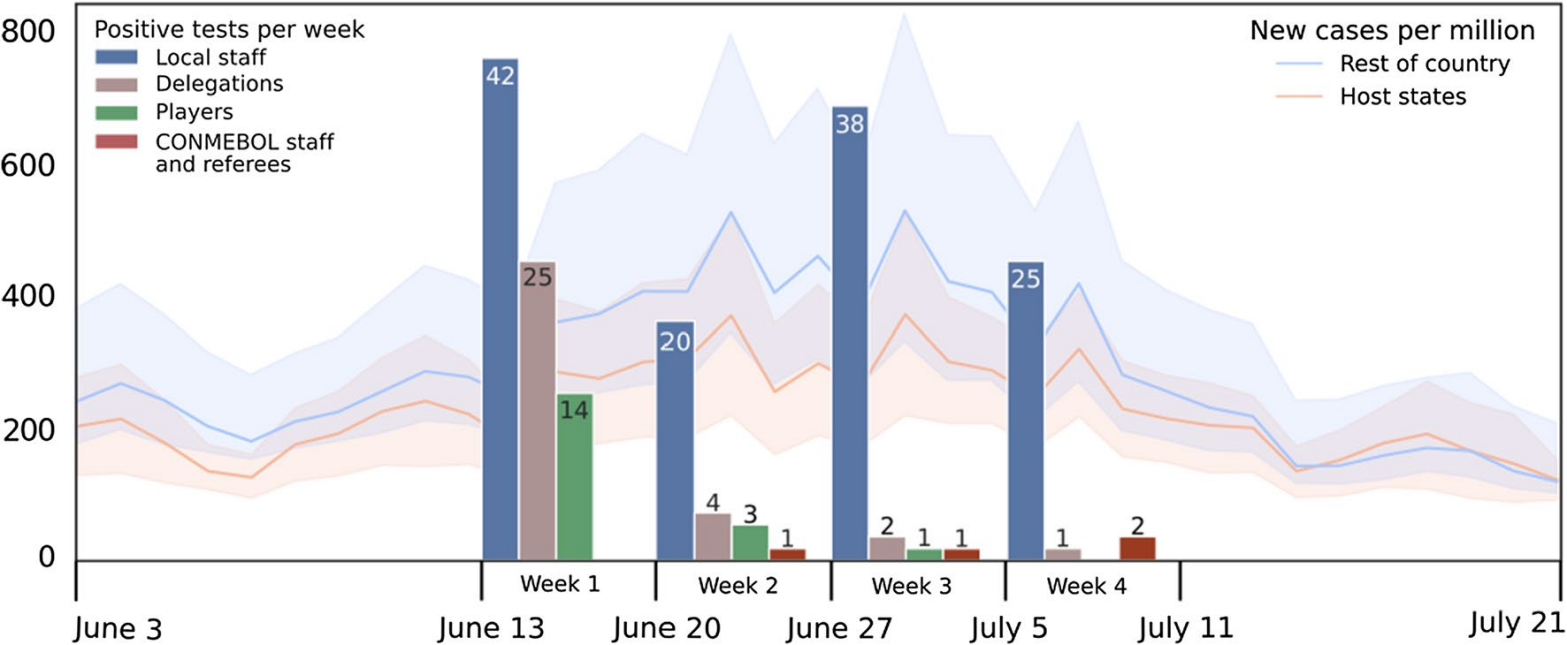
Brazilian epidemiological scenario: The solid lines show the mean number of new COVID-19 cases per million, orange for host states and blue for all other states. Shaded areas represent the 95% confidence interval of cases per million on the corresponding date. Copa América testing results: Bars represent the number of positive tests in the corresponding week of the tournament per group. Note that positive cases in the first week were likely to have originated prior to the start of the tournament

CONMEBOL accredited over 700 persons for Copa América: 250 players in 10 national teams, 210 managers and technical support staff for each team (hereafter referred to as delegations), 250 locally hired staff, and 40 referees and CONMEBOL international staff. Four cities were selected to host the tournament's 28 games. Brasilia and Rio de Janeiro each hosted eight matches, Goiânia seven and Cuiabá five. Cities hosting the tournament were located in federal states with rapidly accelerating contagion rates (Fig. 1).

Public attendance was not allowed except in the final match at the Estádio do Maracanã in Rio de Janeiro, with attendance limited to 10% of the available capacity (~ 7800 people). The mechanics of Copa América 2020 remained otherwise unchanged from its pre-pandemic tournaments (see electronic supplementary material, ESM 1).

### Protocols and Testing Schedule

Three sanitary bubbles were created for groups travelling to Brazil. CONMEBOL international staff and referees were grouped into a common bubble for logistic purposes. Players and their corresponding delegations were kept in separate bubbles since delegations have intrinsically more contact with other bubbles. Importantly, players travelled from over 30 countries to play for their national teams, preventing earlier bubbling. Local staff were not included in the bubbling.

Signs displaying mandatory preventive measures were prominently displayed and hand sanitiser was made available throughout the stadiums. Foreign accredited personnel provided a negative RT-qPCR (real-time polymerase chain reaction) test before travel, and Brazilian regulators required a further RT-qPCR test within 48 h of arrival in the country.

CONMEBOL required RT-qPCR tests for all persons entering the stadiums at any point during the tournament. Samples had to be taken no more than 48 h before processing and all samples were processed in designated laboratories in each host city. Persons with positive test results were required to isolate in their provided accommodations for a minimum of 10 days and until symptom-free for 4 days. Local staff who tested positive were required to follow Brazilian state and federal requirements.

Testing data were collected from all tests after arrival in Brazil between June 13 and July 10, 2021; no follow-up studies were conducted after the end of the tournament. A total of 28,772 tests were performed, with an average of 1027.5 tests for each of the 28 matches. Sequences were obtained for 26 positive samples identifying P1, VOC Gamma GR/501Y.V3 and B.1.621 (VOI) variants, predominant at the time in South America (see ESM 2).

## Findings and Discussion

Cases grew rapidly in both host and non-host states during the first 2 weeks of competition, reaching 600 cases per million in parts of the country. This elevated rate continued during the remaining 2 weeks of the tournament (Fig. 1).

COVID’s high rate of asymptomatic cases together with Copa América’s compulsory testing prevented meaningful comparisons of incidence rates between the general population and the tournament (see ESM 3) [8–14]. Therefore, we analysed the number of positive tests within each group of accredited personnel.

Of the 179 positive tests reported during the tournament, we estimate that 98 were the product of exposure during Copa América. This estimate is based on the exclusion of positive tests reported during the first week of the tournament (see Fig. 1). Games during this first calendar week of the tournament were held 5 days apart, on June 13 and June 18. Considering the accuracy of Rt-qPCR early after exposure to the virus, it is reasonable to assume that positive cases detected during the first week are the product of contagion occurring prior to the beginning of Copa América (see ESM 4) [13, 15]. Importantly, the compulsory testing during Copa América prevented any infection occurring during the first week from remaining undetected during the second week of the tournament.

Cases in bubbles decreased during the tournament, showing protocols were effective in preventing uncontrolled spread. Considering the unfavourable local epidemiological scenario in Brazil, the bubbles were effective in isolating the tournament from the rest of the country (Fig. 1). Two cases were detected in the CONMEBOL staff and referees bubble during the final week. Protocols were followed and those affected were promptly isolated to avoid further spread.

To better understand the risks involved for local staff, and by extension the communities they live in, we analysed whether contagion in this group exceeded expectations (see ESM 3). Because direct comparison of incidence rates was not feasible due to Copa América’s compulsory testing programme, we established a baseline for contagion based on seroprevalence studies conducted at the time in Brazil [9, 16]. Seroprevalence varied significantly with estimates for 2021 ranging from 17 to 35%, with large variations observed across the country [9, 17–21] (see ESM 4). The 83 positive cases detected from the second week of the tournament onwards represent 33.2% of local staff. This infection rate was comparable to the observed seroprevalence in Brazil at the time, suggesting that the risk for members of the local staff was not in excess of what they were exposed to in the general population.

## Conclusion

These data provide further insight into the COVID-19 related risks involved in professional competition. The data suggest that employment in Copa América did not increase the risk of infection for members of the locally hired staff. Furthermore, it shows that appropriate protocols can be effective in preventing infection among players and foreign staff during the tournament.

The CONMEBOL Copa América experience shows that even in adverse epidemiological conditions, thorough preparation, effective execution and compliance verification can allow professional sporting competitions to take place without undue risk of COVID-19 infection to staff and communities.

## Supplementary Information

Below is the link to the electronic supplementary material.

Supplementary file1 (DOCX 48 kb)

## References

[1] Carmody S (2020). When can professional sport recommence safely during the COVID-19 pandemic? Risk assessment and factors to consider. Br J Sports Med.

[2] McCloskey B (2020). Mass gathering events and reducing further global spread of COVID-19: a political and public health dilemma. The Lancet.

[3] Schinke R (2020). Sport psychology services to high performance athletes during COVID-19. Int J Sport Exerc Psychol.

[4] Wong AY-Y (2020). Impact of the COVID-19 pandemic on sports and exercise. Asia-Pac J Sports Med Arthrosc Rehabil Technol.

[5] Meyer T (2021). Successful return to professional men’s football (soccer) competition after the COVID-19 shutdown: a cohort study in the German Bundesliga. Br J Sports Med.

[6] Schumacher YO (2021). Resuming professional football (soccer) during the COVID-19 pandemic in a country with high infection rates: a prospective cohort study. Br J Sports Med.

[7] Gualano B, Brito GM, Pinto AJ (2022). High SARS-CoV-2 infection rate after resuming professional football in São Paulo, Brazil. Br J Sports Med.

[8] McAloon C (2020). Incubation period of COVID-19: a rapid systematic review and meta-analysis of observational research. BMJ Open.

[9] Middelburg RA, Rosendaal FR (2020). COVID-19: How to make between-country comparisons. Int J Infect Dis.

[10] Hasell J, Mathieu E, Beltekian D (2020). A cross-country database of COVID-19 testing. Sci Data.

[11] Wu SL, Mertens AN, Crider YS (2020). Substantial underestimation of SARS-CoV-2 infection in the United States. Nat Commun.

[12] Ma Q, Liu J, Liu Q (2021). Global percentage of asymptomatic SARS-CoV-2 infections among the tested population and individuals with confirmed COVID-19 diagnosis: a systematic review and meta-analysis. JAMA Netw Open.

[13] Kucirka LM (2020). Variation in false-negative rate of reverse transcriptase polymerase chain reaction-based SARS-CoV-2 tests by time since exposure. Ann Intern Med.

[14] Woloshin S, Patel N, Kesselheim AS (2020). False negative tests for SARS-CoV-2 infection—challenges and implications. N Engl J Med.

[15] Zhang Z (2021). Insight into the practical performance of RT-PCR testing for SARS-CoV-2 using serological data: a cohort study. Lancet Microbe.

[16] Vial P (2022). Seroprevalence, spatial distribution, and social determinants of SARS-CoV-2 in three urban centers of Chile. BMC Infect Dis.

[17] Hallal PC (2020). SARS-CoV-2 antibody prevalence in Brazil: results from two successive nationwide serological household surveys. Lancet Glob Health.

[18] Silveira MF (2020). Population-based surveys of antibodies against SARS-CoV-2 in Southern Brazil. Nat Med.

[19] Ioannidis JPA (2021). Infection fatality rate of COVID-19 inferred from seroprevalence data. Bull World Health Organ.

[20] Barros AJD (2021). Population-level seropositivity trend for SARS-Cov-2 in Rio Grande do Sul, Brazil. Rev Saúde Públ.

[21] Núñez-Zapata SF (2021). High seroprevalence for SARS-CoV-2 infection in South America, but still not enough for herd immunity. Int J Infect Dis.

