# Supplementary material for: Safety of International Professional Sports Competitions During the COVID-19 Pandemic: The Association Football Experience
Source: Sports Med. 2022 Sep 27;53(4):765–8. doi: 10.1007/s40279-022-01763-3 (PMC9514706; doi:10.1007/s40279-022-01763-3)
Supplement: Supplementary file 1 — Supplementary file1 (DOCX 48 kb) [file 40279_2022_1763_MOESM1_ESM.docx]

**Supplementary material to:**

Safety of International Professional Sports Competitions during the COVID-19 Pandemic: The Association Football Experience.

Horacio Caniza PhD*. Universidad Paraguayo Alemana de Ciencias Aplicadas. San Lorenzo, Paraguay

Francisco Forriol MD*. Universidad San Pablo-CEU. Madrid, España

Osvaldo Pangrazio MD. CONMEBOL Confederación Sudamericana de Fútbol, Luque, Paraguay

Mario Gil-Conesa MD.MPH. Universidad de Navarra. Pamplona, España

[**Supplementary material 1. Mechanics of Copa America**](#_ryrvhq497jex) **1**

[**Supplementary material 2. Protocols**](#_cvwveack5ywe) **2**

[Evaluation of the individual protocol interventions](#_wqz564pvf8dq) 5

[**Supplementary material 3. Caseload comparison and compulsory testing**](#_m1rbzad6dq4g) **5**

[RT-qPCR false negatives and local staff](#_iub3um8d6zab) 6

[**Supplementary material 4. Testing and positivity in local staff**](#_llvvlssnpxbt) **6**

[**References**](#_46dzg7zgdmb5) **8**

# Supplementary material 1. Mechanics of Copa America

Copa América started June 13 2021 and ended July 10 2021. The tournament consists of a round-robin group stage of five teams each. Argentina, Bolivia, Chile, Paraguay and Uruguay make up the southern group, and Brazil, Colombia, Ecuador, Perú and Venezuela are the northern group. The top four teams from each group advance to the quarterfinals. The four winners of the quarterfinals advance further to the semi-finals, the winners of which proceed to the final. A match between the losers of the semi-finals is played to determine the 3rd and 4th place

CONMEBOL selected stadiums based on technical requirements for the matches, which included but were not limited to, the quality of the pitch and available facilities. The stadiums to host the tournament's 28 games were:

- Estádio Nilton Santos and Estádio do Maracanã in the city of Rio de Janeiro in the state of Rio de Janeiro, 8 games.
- Arena Pantanal in the city of Cuiabá in the state of Mato Grosso, 8 games
- Estádio Olímpico in the city of Goiânia in the state of Goiás and, 7 games.
- Estádio Nacional Mané Garrincha in the federal district of Brasília 8 games.

The tournament spanned 4 calendar weeks.

- During the first week, 8 matches were played. Two matches were held on the 13th, 2 on the 14th, 2 on the 17th and 2 on the 18th. All ten teams played during the first week.
- During the second week, 8 matches were played. Two on the 20th, 2 on the 21st, 2 on the 23rd and 2 on the 24th. All ten teams played during the second week.
- During the third week, 8 matches were played. Two on the 27th of June, 2 on the 28th of June, 2 on July 2nd and 2 on July 3rd. All ten teams played during the third week.
- The final week saw 4 matches being played. One on the 5th of July, one on the 9th of July, and the final match of the tournament on July 10. 4 teams played during the fourth week.

Stadiums were selected based on technical requirements established by CONMEBOL. Audiences were not allowed, with exception of the final match, hosted in the Maracanã stadium. Capacity was limited to 10% of the available seats, or about 7800 people, all of whom were required to provide negative Rt-qPCR tests.

CONMEBOL accredited 750 persons for the duration of the tournament: 250 players, 210 managers and technical support staff for each national team, 250 members of staff hired locally in Brazil and 40 referees and CONMEBOL staff.

# Supplementary material 2. Protocols

48 hours prior to the flight to Brazil, all persons had to provide a negative RT-qPCR test. and no person with a positive result was allowed to fly. Positive tests were studied individually in order to account for people who could prove that they had had COVID in the previous 3 months. Brazilian regulations required a RT-qPCR test immediately upon arrival.

If a positive case was found, immediate confinement was implemented, and each hotel had an area prepared for this purpose. The confinement established according to the protocol was total isolation for ten days, leaving on day 11, provided that there had been no symptoms for at least the previous four days.

Tests were required for each match for all members of the involved delegation. A total of 28,772 tests were conducted on the 750 accredited personnel. On average 1027.5 for each of the 28 matches in the tournament. As was common practice in Brazil, antigen tests were required on all accredited personnel every five days. Positive results from antigen tests required a confirmatory RT-qPCR test for validation. We do not analyse antigen test results in this work.

Media professionals, some of whom entered the immediate match area of the stadiums, were required to present a certificate of a negative RT-qPCR, carried out a maximum of 48 hours before the match. Test results for media personnel are not reported in this work.

Three health bubbles were implemented:

1. Delegations
2. Players
3. CONMEBOL staff and referees

Bubbles were made to correspond with groups that had foreign members. The main objective of the bubble was to better confine any potential outbreaks through the limitation of social contacts between the bubbles. Members of distinct bubbles were prevented from socialising, an important consideration due to the fact that players, referees and delegation members are well acquainted with one another. Travel arrangements were made to prevent any overlap between the bubbles. Notably, national teams are composed of professionals who play in over 50 countries around the world.

**Supplementary Table 1** summarises the recommended protocols to be implemented during training in Brazil and during competition. Special considere

***Supplementary Table 1.*** *COVID-19 protocol during the competition, for delegations*

|  | Delegations and players | Referees | Persons in immediate match zone |
| --- | --- | --- | --- |
| Test | All RT-qPCR(-) | All RT-qPCR(-) | All RT-qPCR(-) |
| Test periodicity | Test every five days (Antigens)  48 h before the match RT-qPCR | Test every five days (Antigens)  48 h before the match RT-qPCR | 48 h before the match RT-qPCR |
| Travel | Plane: CONMEBOL Protocol  Bus: CONMEBOL Protocol | Plane: CONMEBOL Protocol  Bus: CONMEBOL Protocol | Plane: CONMEBOL Protocol  Bus: CONMEBOL Protocol  Transfers to the stadium in a private vehicle or van with few people. |
| Visits | Restricted: virtual  Relatives: RT-PCR(-) | Restricted: virtual | Restricted: virtual |
| Meals | Buffet | Buffet | Buffet |
| Hotel | Avoid contact with hotel staff  Employees in contact with equipment required RT-qPCR(-) | Avoid contact with hotel staff  Employees in contact with referees RT-PCR(-) | Avoid contact with hotel staff |
| Remarks | Comply with Brazilian health regulations  Comply with airline regulations | Comply with Brazilian health regulations  Comply with airline regulations | Comply with Brazilian health regulations  Comply with airline regulations |

For the final, the gates of the Maracana stadium were opened to 7800 guests. Negative RT-qPCR tests were required for ingress. The tickets were non-transferable, and a register of the spectator's name, surname, ID number, email, telephone number and seat number was kept in case of reported outbreaks. As required by Brazilian regulations, all persons had their temperature checked at the gate. No records of these temperatures were kept.

They were shown the “code of conduct”, requiring them to avoid crowds, stay in their seats, not stand up, not carry cheering objects (e.g. *vuvuzela*), wear masks at all times and avoid hugging and greeting others fans when cheering.

During and after the match, measures were taken to ensure minimum contact between spectators and in particular to maintain social distance. General hygiene measures were taken inside the stadium and at the entrances, such as the installation of hand sanitiser dispensers. Security was in charge of ensuring compliance with prevention measures and the management of queues at the gates and restrooms. There were no food and drink stands.

Lastly, CONMEBOL organised a vaccination centre in Paraguay, available to all CONMEBOL delegations and staff who wanted to vaccinate.

## Evaluation of the individual protocol interventions

Evaluation of the individual elements of the protocol was not possible during Copa América. In particular, the bubbling concept was not evaluated in isolation.

While it is reasonable to assume that bubbles are useful, and the evidence seems to point in that direction [WIL, TRN, LEN], it was not possible to isolate their effect in Copa América. Importantly, we are of the opinion that any success is due to the combination of measures implemented and the compliance to these measures, rather than a single individual measure.

# Supplementary material 3. Caseload comparison and compulsory testing

Direct comparisons of contagion rates between populations is a difficult, and many times impossible, problem [RUT]. This problem is multifactorial, one such factor is the chronic underreporting of COVID cases, a problem so complex that it is not limited to lower-income countries [HAS,WU,MA]. The difficulty in comparing caseloads between populations is not related to the size of the population. That is, while the Copa América population is limited to 750 people, the underlying difficulties in comparing caseloads between the different groups within this population remain.

An important factor to consider is that testing was compulsory for all persons involved in the tournament. Testing was, at the time, not compulsory for the general Brazilian population. Between June 13 and July 10 2021, Brazil performed an average of 150.338 tests per day on average in the entire country (0.0007 per person per day) with a total for the period of 3.9 million tests ( 0.018 tests per person) [HAN]. In contrast, Copa América performed 1.37 tests per person per day during the same period, a testing rate about 76 times higher.

Considering that up to 40% of COVID cases are asymptomatic [MA], at-will testing would necessarily result in a lower reported caseload for all those people who do not get tested since they exhibit no symptoms. Furthermore, a compulsory testing programme would also detect positive persons that would otherwise be unwilling or unable to get tested [RUB]. We have chosen to compare the number of positive cases during Copa América with the caseload in Brazil.

Interestingly, host states with the highest incidence [JHU] do not have the highest number of positive tests in the local staff group (**Suppl. Figure 1**)

| 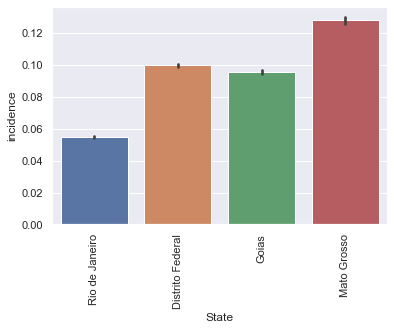 | 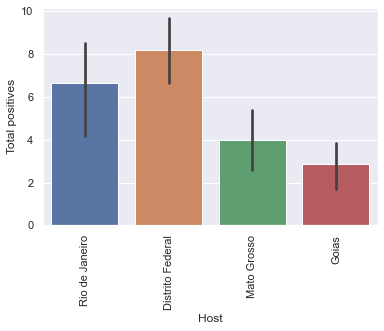 |
| --- | --- |

**Suppl Figure 1.** Comparison of incidence rate in host states and positive tests per group in each host state.

## RT-qPCR false negatives and local staff

Early after exposure, RT-qPCR tests have little to no diagnostic accuracy. Within five days of exposure, false positive rates can climb as high as 67% [WOL,KUC].

Based on this argument, we are able to exclude positive cases from the first week of the tournament. In particular, considering that Goiânia and Rio de Janeiro each hosted two games 5 days apart on June 13 and on June 18. Brasília and Cuiabá also hosted two games each 4 days apart, on June 14 and on June 17.

After considering this period the number of local staff who are likely to have contracted the disease during Copa América is 83.

# Supplementary material 4. Testing and positivity in local staff

Testing and positivity per group were as follows:

- Local staff: 66.2% of all tests and, 69.83% of all positive cases
- CONMEBOL and referees: 17.9% of all tests, 2.23% of all positive cases.
- Delegations: 12.6% of all tests, 17.88% of all positive cases
- Players 5.3% of all tests and 10.06% of all positive cases.

A brief discussion on the local staff is pertinent. This is a particularly important group to study because they constitute “interfaces” between the community and Copa América. To reiterate, these positive cases are also the product of Copa América’s compulsory testing, and are, therefore, not directly comparable to the general population.

Nevertheless, it is important to determine whether the risk to the local staff group was higher than that of the general population in which they live. One way to estimate whether local staff was exposed to higher risk than the general population is to compare the positivity rate with reported seroprevalence studies. While these studies vary greatly, they aim to measure the presence of antibodies against SARS-CoV-2 from blood samples from randomly selected people in the population. The presence of antibodies can signify that a person has been infected at some point before the test was performed [HAL].

Comparing seropositivity, i.e. the fraction of people with antibodies, with the positivity rate product of the compulsory testing scheme, would show whether the risk was elevated for local staff during Copa América.

Hallal et al. have compiled a large number of seropositivity studies performed in Brazil. Samples were taken in two stages from May 14 to May 21 and June 4 to June 7 of 2020, at the beginning of the pandemic. The study reports varying seropositivity, namely in Cuiabá (state of Mato Grosso) between 4.2% and 6.3%, in Rio de Janeiro (state of Rio de Janeiro) at 2.4% and 7.5%, and both Brasilia (federal district, located within the state of Goiás) and Goiânia (state of Goiás) 0% seropositivity [HAL]. By September of 2020 parts of the federal state of Goiás, where Brasilia and Goiânia are located, reported seropositivity levels of up to 8.1%. Barros et al. reported an increase of almost 18% between April 2020 and February 2021 (April 2020 was 0.07%, increasing to 10.0% in February 2021, and to 18.2% in April 2021) in the state of Rio Grande do Sul. Other states report a similar increase in seroprevalence.

The general trend in seropositivity across Brazil shows an increase towards the end of 2020, with levels between 13.5% to 35% [COS, ION, LAL, BAR]. The 83 positive tests reported in the local staff group represent an incidence rate of 33.2% comparable to observed seroprevalence in Brazil at the time. These results suggest that the risk for members of the local staff was not in excess of what they were exposed to in the general population

From this brief analysis, we cannot conclude that the risk was lower than the community or that Copa América afforded any protection against the virus. Rather, we limit ourselves to state that local staff were not exposed to a higher risk than they would in the community

By June 2021, when Copa América started, about 20% of the Brazilian population was vaccinated impeding further seroprevalence studies. However, analysing this positivity rate in relation to the host city’s characteristics such as the number of inhabitants, urban structures and population density, could provide further insight into the particularities of the effect of a tournament of this scale on the local population.

# References

[MA] Ma Q, Liu J, Liu Q, et al. Global Percentage of Asymptomatic SARS-CoV-2 Infections Among the Tested Population and Individuals With Confirmed COVID-19 Diagnosis: A Systematic Review and Meta-analysis. JAMA Netw. Open. 2021;4(12):e2137257. doi:10.1001/jamanetworkopen.2021.37257

[RUB] Rubin R. First It Was Masks; Now Some Refuse Testing for SARS-CoV-2. JAMA. 2020;324(20):2015–2016. doi:10.1001/jama.2020.22003

[RUT] Rutger A. Middelburg, Frits R. Rosendaal, COVID-19: How to make between-country comparisons, International Journal of Infectious Diseases,Volume 96, <https://doi.org/10.1016/j.ijid.2020.05.066>.

[HAS] Hasell, J., Mathieu, E., Beltekian, D. et al. A cross-country database of COVID-19 testing. Sci Data 7, 345 (2020). <https://doi.org/10.1038/s41597-020-00688-8>

[WU] Wu, S.L., Mertens, A.N., Crider, Y.S. et al. Substantial underestimation of SARS-CoV-2 infection in the United States. Nat Commun 11, 4507 (2020). <https://doi.org/10.1038/s41467-020-18272-4>

[WIL] Willem, Lander, et al. "The impact of contact tracing and household bubbles on deconfinement strategies for COVID-19." Nature communications 12.1 (2021): 1-9.

[TRN] Trnka, Susanna, and Sharyn Graham Davies. "Blowing bubbles: COVID-19, New Zealand’s bubble metaphor, and the limits of households as sites of responsibility and care." COVID-19. Routledge, 2020. 167-183.

[LEN] Leng, Trystan, et al. "The effectiveness of social bubbles as part of a Covid-19 lockdown exit strategy, a modelling study." Wellcome open research 5 (2020).

[HAL] Hallal, Pedro C., et al. "SARS-CoV-2 antibody prevalence in Brazil: results from two successive nationwide serological household surveys." The Lancet Global Health 8.11 (2020): e1390-e1398.

[BAR] Barros, Aluísio J D et al. “Population-level seropositivity trend for SARS-Cov-2 in Rio Grande do Sul, Brazil.” Revista de saude publica vol. 55 78. 22 Nov. 2021, doi:10.11606/s1518-8787.2021055004075

[COS] Costa, Lourenço Faria, et al. "Seroprevalence of SARS-CoV-2 in southwest Goiás, Brazil, 2020: a population-based cross-sectional serological study." One Health & Implementation Research 1.1 (2021): 31-42.

[ION] Ioannidis JPA. Infection fatality rate of COVID-19 inferred from seroprevalence data. Bull World Health Organ. 2021;99(1):19-33F. doi:10.2471/BLT.20.265892

[KUC] Kucirka, Lauren M et al. “Variation in False-Negative Rate of Reverse Transcriptase Polymerase Chain Reaction-Based SARS-CoV-2 Tests by Time Since Exposure.” Annals of internal medicine vol. 173,4 (2020): 262-267. doi:10.7326/M20-1495

[WOL] Woloshin, Steven, Neeraj Patel, and Aaron S. Kesselheim. "False negative tests for SARS-CoV-2 infection—challenges and implications." New England Journal of Medicine 383.6 (2020): e38.

[HAN] Hannah Ritchie, Edouard Mathieu, Lucas Rodés-Guirao, Cameron Appel, Charlie Giattino, Esteban Ortiz-Ospina, Joe Hasell, Bobbie Macdonald, Diana Beltekian and Max Roser (2020) - "Coronavirus Pandemic (COVID-19)". Published online at OurWorldInData.org. Retrieved from: 'https://ourworldindata.org/coronavirus' [Online Resource]
